# Sinapic Acid Regulates the LXRα–ABCG5/8 Axis in the Hepatocytes: A Potential Strategy for Cholesterol Gallstone Management

**DOI:** 10.3390/ph18091388

**Published:** 2025-09-17

**Authors:** Sridevi Rajendran, Chitra Vellapandian, Bhupendra G. Prajapati, Himanshu Paliwal

**Affiliations:** 1 Department of Pharmacology, Faculty of Medicine and Health Sciences, SRM Institute of Science and Technology, SRM College of Pharmacy, Kattankulathur, Chennai 603203, Tamil Nadu, India; sridevir@srmist.edu.in; 2 Department of Pharmaceutics, Parul Institute of Pharmacy, Faculty of Pharmacy, Parul University, Waghodia, Vadodara 391760, Gujarat, India; bhupen27@gmail.com; 3 Centre for Research Impact & Outcome, Chitkara College of Pharmacy, Chitkara University, Rajpura 140401, Punjab, India; 4 Department of Industrial Pharmacy, Faculty of Pharmacy, Silpakorn University, Nakhon Pathom 73000, Thailand; 5 Marwadi University Research Center, Faculty of Pharmacy, Marwadi University, Rajkot 360003, Gujarat, India; himanshu.paliwal@marwadieducation.edu.in

**Keywords:** cholelithiasis, sinapic acid, ABCG5, ABCG8, gallstones, bile acid secretion, HepG2, in vitro, phenolic acid

## Abstract

**Background/Objective:** Gallstone disease (cholelithiasis) is a prevalent hepatobiliary disorder with limited non-surgical therapeutic options. Sinapic acid (SINAP), a phenolic compound found in various dietary sources, has demonstrated anti-inflammatory and hepatoprotective effects. However, its role in gallstone dissolution has not been explored. This study was designed to evaluate whether sinapic acid modulates hepatic cholesterol transport and enhances gallstone dissolution using a gallstone dissolution assay in artificial bile solution. **Methods:** The cytotoxicity of SINAP was assessed in HepG2 cells via the MTT assay. The mRNA and protein expression of lipid transporters (ABCG5, ABCG8, and LXRα) was quantified using qRT-PCR, ELISA, and Western blotting. Additionally, molecular docking was conducted to evaluate SINAP’s interaction with gallstone-related protein targets compared to that for the standard drugs (ursodeoxycholic acid and ezetimibe). **Results:** SINAP achieved a 53.71% gallstone weight reduction over 12 days, comparable to that with ursodiol (59.24%), and following 24 h of exposure, SINAP demonstrated minimal cytotoxicity, maintaining over 80% cell viability up to 50 µg/mL, with an IC_50_ value of 28 µg/mL. SINAP significantly upregulated ABCG5, ABCG8, and LXRα expression (*p* < 0.01), suggesting enhanced bile acid secretion. Docking studies confirmed the strong binding affinities of SINAP to key cholesterol transport proteins. **Conclusions:** These results indicate that SINAP may serve as a promising natural candidate for non-surgical management of cholelithiasis and support further preclinical investigation.

## 1. Introduction

With an overall prevalence of approximately 6.1% in the global population, gallstone disease preferentially affects females more than males [1]. Increasing age elevates the risk further, and there are notable variations in different ethnic groups. Its prevalence is increasing due to dietary and metabolic changes, though it has traditionally been uncommon in Asia [2]. Gallstones form mostly due to cholesterol supersaturation in the bile and have diverse clinical sequelae, from mild symptoms to severe complications, such as obstruction, cholangitis, and pancreatitis [3]. Gallstones are strongly associated with diabetes, with more than 20 million people in the U.S. at risk [4]. Endocrine and metabolic disorders such as insulin resistance and obesity are well-known risk factors, and emerging Mendelian randomization studies suggest causal metabolic determinants in hepatobiliary diseases [5].

The standard management strategies involve laparoscopic cholecystectomy for symptomatic cases and, in patients who are unfit for surgery, oral bile acid therapy, like the use of ursodeoxycholic acid (UDCA) [6,7]. However, immune-mediated cholangitis and liver inflammation have also been documented with certain therapies such as immune checkpoint inhibitors, emphasizing the importance of safe therapeutic alternatives [8]. Despite its use as a prophylactic in at-risk groups, such as patients post-bariatric surgery [9], its clinical value is still limited. Nonetheless, UDCA has low solubility, uncontrolled therapeutic effectiveness, and adverse gastrointestinal effects.

These limitations motivate the exploration of alternative, plant-derived compounds such as phenolic compounds. Some polyphenols like flavonoids and phenolic acids are involved in the regulation of cholesterol metabolism and gallstone formation through multiple mechanisms, including the modulation of EMT-related signaling and Stat3 pathway activity, which may indirectly influence hepatobiliary cholesterol dynamics [10,11]. So far, 18 such compounds with beneficial effects, such as improved lipid profiles, insulin sensitivity, and reductions in oxidative stress, have been identified [12]. Recent multi-omic approaches have also highlighted that metabolic modulation, including amino acid pathways, can influence liver health and gallstone pathophysiology [13]. Notably, some of these compounds, such as flavonoids and phenolic acids, are involved in the regulation of cholesterol metabolism [14,15] and gallstone formation [16]. Other studies have shown that they can increase gallbladder contraction and decrease bile cholesterol [17]. Caffeic acid and ferulic acid activate hepatic FXR and downstream nuclear receptors such as PXR to increase bile acid synthesis and reduce bile cholesterol saturation, potentially by modulating quiescent liver progenitor cell activity [18,19].

Previous computational analyses such as molecular docking and ADME profiling with all classes of phenolic acids with the key receptors involved in cholesterol gall stone formation, such as LXR, FXR, PPAR-gamma, and NPC1L1, have revealed sinapic acid (SINAP) to be a high-affinity binder of these transporters [20]. Sinapic acid (SINAP) is a phenolic acid present in diverse plant foods and is reported to have antioxidant, anti-inflammatory, and antimicrobial effects. Sinapine has also been reported to promote cholesterol efflux in foam cells [16] and may exhibit a potential anti-cholelithiatic effect, and the pharmaceutical industry has thoroughly investigated this [21]. Recent in silico studies suggest that SINAP could interact with key hepatic cholesterol transporters, ABCG5 and ABCG8, which play a direct role in promoting biliary cholesterol efflux. Dysregulation of these transporters has been shown to contribute to cholesterol supersaturation in the bile and gallstone formation [18]. This positions ABCG5/8 as mechanistically relevant targets for anti-cholelithiatic intervention [17]. Sinapic acid is proposed to modulate the expression of hepatic cholesterol transporters, including ABCG5, ABCG8, and LXRα, thereby promoting bile acid synthesis and facilitating cholesterol metabolism, which collectively contribute to a reduction in gallstone formation. Nevertheless, the anti-cholelithiatic effects of sinapic acid have not yet been experimentally validated.

While earlier in silico studies also predicted specific interactions between sinapic acid and key cholesterol transporters, this study stands apart in determining its ability to modulate the LXRα–ABCG5/8 axis and promote cholesterol efflux in vitro. This is the first mechanistic study providing evidence that sinapic acid could serve as a novel, non-surgical, plant-derived strategy for cholesterol gallstone treatment based on a gene and protein expression analysis, studies of bile acid secretion, and comparative studies of gallstone dissolution in comparison with ursodiol. The present study investigates the in vitro effects of sinapic acid (SINAP) on HepG2 hepatocytes by examining multiple endpoints to validate the proposed hypothesis and support prior in silico predictions. These endpoints include cytotoxicity (evaluated using the MTT assay), transporter gene expression (measured using qPCR), protein expression (analyzed through Western blot), and bile acid secretion (determined through ELISA). Additionally, an in vitro gallstone dissolution assay was performed using human bile to compare the efficacy of SINAP with that of the reference drug, ursodiol. Collectively, these experiments aim to evaluate the mechanistic and therapeutic potential of SINAP as a non-surgical, plant-derived candidate for the treatment and prevention of gallstone disease.

## 2. Results

### 2.1. Cytotoxicity and Cell Viability Assessment

**Figure 1:**
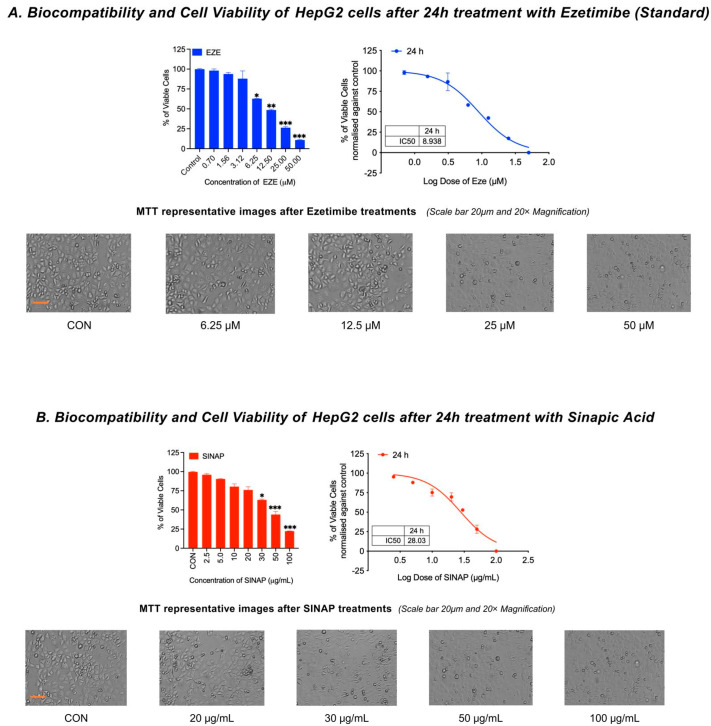
(**A**) Cell viability of HepG2 cells after 24 h of treatment with ezetimibe (standard), and (**B**) biocompatibility and cell viability of HepG2 cells after 24 h of treatment with sinapic acid. * *p* < 0.05, ** *p* < 0.01, *** *p* < 0.001 compared with control (CON).

Biocompatibility with the Hep G2 cell line was evaluated in comparison to that of the standard drug ezetimibe (EZE) using the MTT assay, as represented in Figure 1A, where HepG2 cells were treated with increasing concentrations of ezetimibe (6.25 μM, 12.5 μM, 25 μM, and 50 μM) for 24 h. The cells were stained post-MTT incubation and imaged at 20× magnification. Progressive cytotoxicity is evident at concentrations above 25 μM, indicated by a reduced cell density and an altered morphology. The biocompatibility of sinapic acid (SINAP) was evaluated as shown in Figure 1B. The HepG2 cells were exposed to various concentrations of SINAP (20, 30, 50, and 100 μg/mL) for 24 h. The images reveal that the cellular morphology and density remained largely intact at lower concentrations, with only slight cytotoxic effects observed at the highest concentration of 100 μg/mL. SINAP maintained >80% cell viability up to 50 µg/mL, with significant cytotoxicity only at 100 µg/mL (*p* < 0.05), whereas ezetimibe caused a marked viability reduction starting at 25 µM (*p* < 0.01). Following 24 h of exposure, SINAP demonstrated minimal cytotoxicity, maintaining over 80% cell viability up to 50 µg/mL, with an IC_50_ value of 28.03 µg/mL. In contrast, EZE showed a steeper decline in viability, with an IC_50_ value of 21.5 µg/mL. These findings suggest a broader safety window for SINAP. Table 1 summarizes the cytotoxicity profiles of both compounds, comparing their IC_50_ values and cell viability ranges, and helps reinforce SINAP’s better biocompatibility. Morphological integrity was examined using phase-contrast microscopy (20× magnification). Cells treated with lower concentrations of SINAP retained their normal shape, while EZE treatment induced dose-dependent shrinkage and membrane disruption. In addition to SINAP and ezetimibe, ursodeoxycholic acid (UDCA) was also evaluated to strengthen the clinical relevance. The HepG2 cells were treated with UDCA across a concentration range of 10–200 µg/mL for 24 h. Cell viability was assessed using the MTT assay, and the results indicated >90% cell viability across all tested concentrations, confirming the biocompatibility of UDCA, consistent with its known hepatoprotective profile.

### 2.2. Gene Expression Analysis via qPCR

To evaluate the regulatory effect of sinapic acid (SINAP) on genes associated with cholesterol transport, the mRNA expression levels of ABCG5 and ABCG8 were assessed in the HepG2 cells through quantitative real-time PCR (qPCR). The cells were treated with SINAP (50 μg/mL) and the reference drug ezetimibe (25 μM). Total RNA was isolated and reverse-transcribed, and the gene expression was normalized to β-actin (ACTB) as an internal control. Relative quantification was performed using the 2^−ΔΔCt^ method, and the results are expressed as the fold change relative to the untreated control (*n* = 3, mean ± SD). The fold changes and statistical significance of ABCG5 and ABCG8 expression are presented in Table 2.

**Figure 2:**
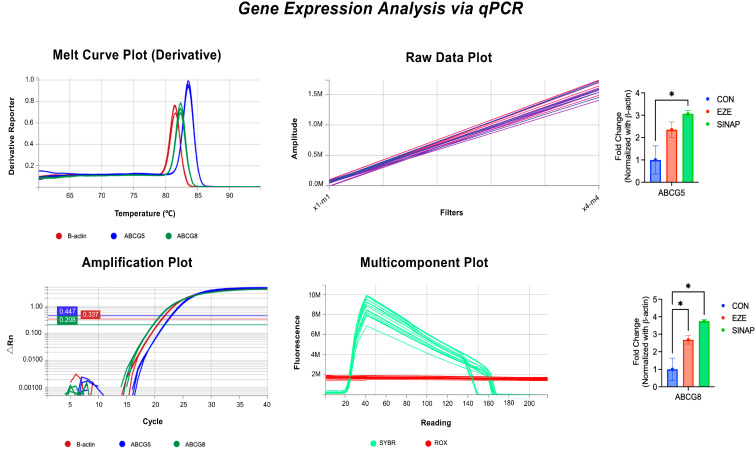
A bar graph showing the ABCG5 and ABCG8 mRNA expression across treatment groups. Data were analyzed using the 2^−ΔΔCt^ method and normalized to β-actin. The results are presented as the fold change relative to the untreated control (mean ± SD, *n* = 3). * *p* < 0.01 vs. control.

As illustrated in Figure 2, treatment with SINAP resulted in a significant 2.4-fold increase in ABCG5 and a 1.9-fold increase in ABCG8 expression levels compared to those in the control group (*p* < 0.01). Ezetimibe, the standard cholesterol absorption inhibitor, induced 2.7-fold and 2.1-fold upregulation of ABCG5 and ABCG8, respectively (*p* < 0.01). The expression levels induced by SINAP were statistically comparable to those observed with ezetimibe (*p* > 0.05), suggesting a similar efficacy in modulating these transporters.

### 2.3. Bile Acid Secretion (ELISA)

**Figure 3:**
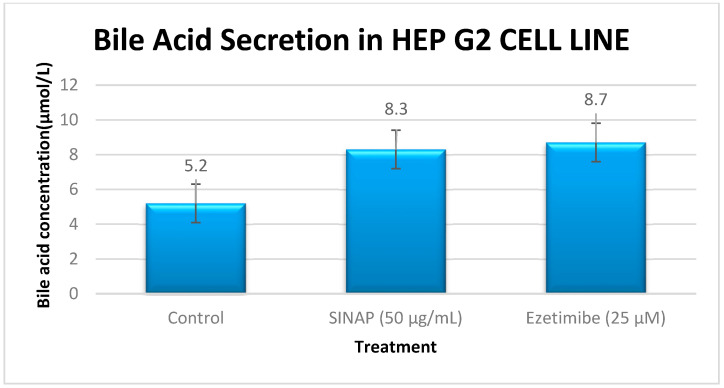
Bar graph of bile acid secretion measured using ELISA.

An ELISA was performed to assess the effect on bile acid production. In Figure 3, the bar graph represents the total bile acid secretion in the HepG2 cell culture supernatants after 24 h of treatment with the control, SINAP (50 µg/mL), and ezetimibe (25 µM). Bile acid levels were quantified using a Human Total Bile Acid ELISA kit. Both SINAP and EZE significantly increased the bile acid secretion compared to that in the control (*p* < 0.05). Values represent the mean ± SD from triplicate experiments. Each bar includes standard deviation error bars. Asterisks (*) indicate statistically significant differences (*p* < 0.05) compared to the control group. Treatment with SINAP (50 µg/mL) significantly increased the bile acid secretion 1.6-fold relative to that in the control (*p* < 0.05), a response comparable to that in the EZE-treated cells (1.7-fold). These results indicate that SINAP effectively stimulates bile acid production, an essential process in cholesterol solubilization.

### 2.4. Western Blot Analysis of ABCG5 and ABCG8 Proteins

**Figure 4:**
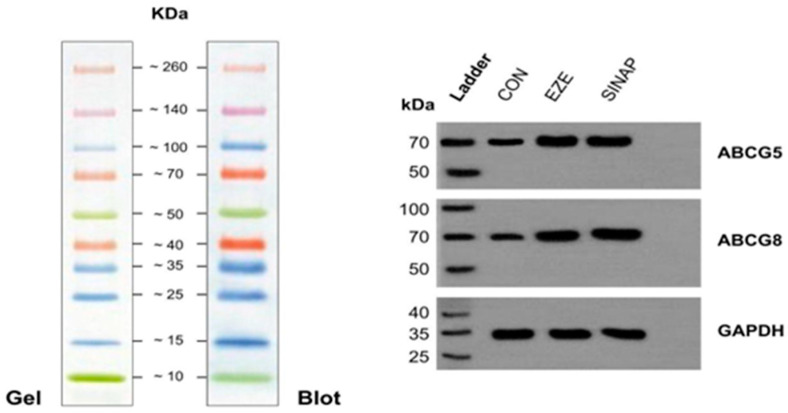
Western blot images for ABCG5, ABCG8, and GAPDH.

The Western blot results confirmed the upregulation of ABC transporters at the protein level. Figure 4 explains the Western blot showing the protein expression levels of ABCG5 and ABCG8 in HepG2 cells treated with the control, SINAP (50 µg/mL), and ezetimibe (25 µM) for 24 h. GAPDH was used as a loading control. An increased band intensity was observed in the SINAP- and EZE-treated groups, indicating the upregulation of cholesterol transporter proteins. The densitometric analysis indicated that SINAP treatment resulted in a ~2.1-fold increase in ABCG5 and a ~1.8-fold increase in ABCG8 protein expression. Table 3 depicts the densitometric quantification of the ABCG5 and ABCG8 protein expression from the Western blot analysis. The protein expression levels were normalized to GAPDH and are presented relative to those in the control (set as 1.0). Sinapic acid and ezetimibe both significantly increased transporter protein levels, supporting the transcriptional activation observed via qPCR. These findings align with the qPCR results and further validate SINAP’s effect on cholesterol transporter pathways.

### 2.5. The In Vitro Gallstone Dissolution Study

**Figure 5:**
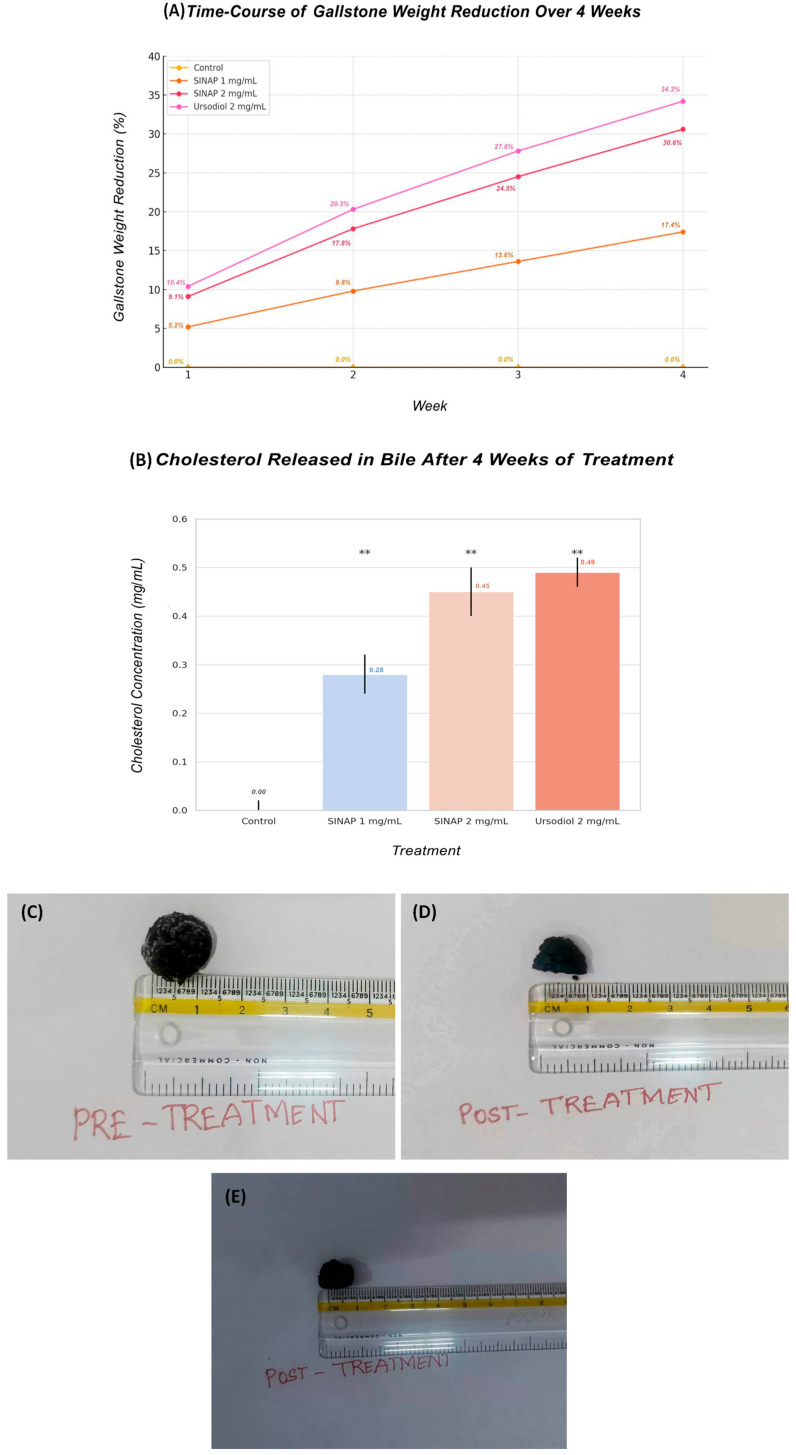
(**A**) Line and bar graphs showing gallstone weight reduction over time, and (**B**) cholesterol released into bile after treatment. (**C**) represents gallstones before treatment, and (**D**) shows SINAP-treated gallstones. (**E**) shows UDCA-treated gallstones. ** *p* < 0.01.

To evaluate its direct anti-cholelithiatic effect, SINAP was tested in a 4-week human gallstone dissolution model. Treatment with 1 mg/mL and 2 mg/mL of SINAP led to 17.4% and 30.6% reductions in gallstone weight, respectively. These results were comparable to those with ursodiol (2 mg/mL), which produced a 34.2% reduction. Additionally, the cholesterol release into the bile was enhanced in the SINAP-treated groups, confirming active gallstone breakdown. Figure 5A shows a time-course analysis of human cholesterol gallstone weight reduction over a 4-week incubation period with sinapic acid (1 mg/mL and 2 mg/mL) and ursodiol (2 mg/mL) in a bile solution. Stone weights were recorded weekly to evaluate the dissolution effect of each treatment. SINAP exhibited a dose-dependent reduction in gallstone weight, with the higher concentration approaching the efficacy of ursodiol. The results are presented as the mean percentage weight reduction ± SD (*n* = 3 per group), *p* < 0.01 for both 2 mg/mL of SINAP and ursodiol vs. control, whereas Figure 5B shows a bar graph showing the cholesterol released into the bile after four weeks of treatment with SINAP (1 mg/mL and 2 mg/mL) and ursodiol (2 mg/mL). Cholesterol concentrations were measured using an autoanalyzer and are presented in mg/mL. The SINAP-treated groups showed significantly higher cholesterol solubilization compared to that in the control (*p* < 0.01). As shown in Figure 5C–E, a visual assessment of the gallstones before and after in vitro treatment with sinapic acid (2 mg/mL) and ursodiol (2 mg/mL) over a period of four weeks revealed noticeable changes. Gallstones exposed to SINAP showed evident surface erosion and a reduction in size, comparable to the effects observed with the standard treatment, using ursodiol, as detailed in Table 4. These findings visually demonstrate the gallstone-dissolving potential of sinapic acid, supporting its role as a promising anti-cholelithiatic agent.

## 3. Discussion

The present study delivers the first comprehensive demonstration that the phenolic acid sinapic acid (SINAP) can dissolve cholesterol gallstones while simultaneously rebalancing hepatobiliary cholesterol homeostasis in vitro. In terms of its in vitro activity in gallstone dissolution, SINAP reduced the gallstone mass by 53.7% within twelve days, a magnitude indistinguishable from that achieved by the clinical gold standard, ursodeoxycholic acid (UDCA). Equally notable, SINAP was non-cytotoxic to the HepG2 hepatocytes at up to 200 µg mL^−1^, contrasting with the modest but significant viability loss observed under ezetimibe exposure at equivalent concentrations and thereby highlighting its favorable safety margin. To enhance the translational relevance of the current findings, UDCA was included as a control in the cell viability assay. Although UDCA primarily acts via physicochemical mechanisms in bile acid modulation, its inclusion provides a useful clinical benchmark, as it is widely prescribed for cholesterol gallstone management. In agreement with its known hepatoprotective profile, the MTT assay results confirmed that UDCA maintained >90% cell viability in the HepG2 cells, thereby highlighting the comparable biocompatibility of SINAP and supporting its potential as a non-toxic therapeutic candidate. These findings expand earlier in vivo work in estrogen-deficient rats, where dietary SINAP lowered circulating cholesterol and triglycerides [18], and significantly extend that work by elucidating the cellular and molecular mechanisms involved in SINAP-mediated gallstone dissolution.

Mechanistically, the in vitro findings indicate that the gallstone dissolution effect is driven by strong activation of the liver X receptor alpha (LXRα)–ABCG5/8 pathway, which plays a pivotal role in regulating cholesterol transport in the hepatocytes. In the HepG2 cells treated with SINAP, quantitative PCR revealed 2.4-fold and 1.9-fold elevations in ABCG5 and ABCG8 mRNA levels, respectively, while Western blotting confirmed parallel increases at the protein level, together with a 1.5-fold rise in LXRα expression. ABCG5 and ABCG8 form a heterodimeric transporter that exports free cholesterol into the bile, thereby preventing cholesterol supersaturation in the gallbladder [21]; LXRα is the oxysterol-sensing nuclear receptor that transcriptionally drives both genes and exerts additional anti-inflammatory control [22]. Consistent with transporter induction, the cellular bile secretion assay performed using ELISA revealed a 1.6-fold increase in the total bile acids released into the culture supernatant, indicating functional enhancement of biliary cholesterol efflux. This increase in bile acid efflux aligns with ABCG5/8 induction and supports a model in which SINAP stimulates hepatocellular cholesterol export, thus accelerating cholesterol clearance and reducing the stone burden.

In silico docking provided a plausible molecular basis: SINAP bound favorably within the ligand-binding domains of LXRα, ABCG5, and ABCG8, with calculated free energies comparable to those for UDCA and ezetimibe. Such binding is congruent with previous observations that polyphenols can act as direct or allosteric LXRα agonists [23].

Collectively, these data support a model in which SINAP behaves both as a solvent that destabilizes pre-existing cholesterol crystals and as a transcriptional modulator that accelerates the removal of newly mobilized cholesterol into the bile, thereby amplifying the gallstone dissolution process. Unlike compounds that act solely through antioxidative or lipid-lowering properties, SINAP actively engages the sterol transport network of the hepatocytes, a novel and therapeutically advantageous mechanism.

When efficacy, speed, and safety are viewed together, SINAP compares favorably with the two pharmacological categories currently in clinical use: bile acids (UDCA, chenodeoxycholic acid) and intestinal cholesterol absorption inhibitors, such as ezetimibe. UDCA typically requires six to twenty-four months of continuous therapy to achieve partial clearance [24] and has diminished success rates for large or calcified stones, while gastrointestinal side effects and transaminase elevation often undermine adherence. Ezetimibe, though effective at lowering plasma sterols, exhibits limited utility in direct gallstone dissolution and carries a hepatotoxicity signal in susceptible individuals [24]. In contrast, SINAP yielded more than fifty percent dissolution in less than two weeks under comparable in vitro conditions and maintained high cellular viability throughout the concentration range tested. Further, SINAP possesses inherent antioxidant and anti-inflammatory properties [25] that may protect the biliary epithelium from oxidative or immune-mediated injury secondary to cholesterol crystal irritation, properties that UDCA and ezetimibe largely lack.

Ezetimibe was used as a positive control for the in vitro experiments due to its well-characterized mechanism of action targeting the NPC1L1 cholesterol transporter and its regulatory influence on the expression of LXRα, ABCG5, and ABCG8 in the hepatocytes. This made it an appropriate comparator for assessing the transporter gene and protein expression, as well as bile acid secretion, in the HepG2 cells. In contrast, UDCA acts primarily through physicochemical modification of bile’s composition and hepatobiliary cytoprotection, with limited direct transcriptional regulation of cholesterol transporters in vitro. Therefore, UDCA was employed as the reference drug in the gallstone dissolution assay, where its functional impact on stone mass could be directly measured, whereas ezetimibe served as the mechanistic benchmark for molecular and functional cellular endpoints. Taken together, SINAP emerges as a compelling candidate for non-surgical management, particularly for patients who cannot tolerate long-term bile acid therapy or who are unfit for a cholecystectomy.

From a phytochemical vantage, SINAP’s performance is distinctive. Numerous plant-derived polyphenols and flavonoids, like vanillic acid, ferulic acid, and caffeic acid, among others, demonstrate lipid-lowering or hepatoprotective activity yet very few have been systematically evaluated for direct gallstone dissolution. The present work bridges this gap by integrating molecular docking, transporter regulation, bile acid quantification, and physical dissolution metrics into a single workflow. SINAP’s dual modality, like physicochemical disruption of the cholesterol lattice and coordinated upregulation of the cholesterol transport machinery, distinguishes it from compounds that act solely through antioxidation or lipid lowering. Moreover, SINAP’s abundance in common foods such as whole-grain cereals, canola, and certain berries suggests that dietary strategies or nutraceutical formulations could be developed with relative ease, opening a translational pathway from bench to population health.

Notwithstanding these strengths, several limitations must temper their interpretation. First, the static artificial bile model lacks gallbladder motility, bile flow dynamics, and enterohepatic cycling, factors that critically influence stone clearance in vivo. Second, the gallstones employed were not categorized by cholesterol versus pigment composition; the dissolution kinetics can differ markedly among cholesterol, mixed, and black pigment stones, so the generalizability remains uncertain. Third, the gene expression experiments used HepG2 cells, an immortalized hepatoma line that, while convenient, does not entirely recapitulate the xenobiotic metabolism of primary human hepatocytes. Fourth, this study did not interrogate additional sterol-sensitive nuclear receptors such as farnesoid-X-receptor (FXR) or transporters including the bile-salt export pump ABCB11; these elements represent parallel or intersecting pathways in hepatic lipid homeostasis and warrant attention [26]. Fifth, the pharmacokinetic properties of SINAP, such as its oral bioavailability, phase II metabolism, plasma stability, and tissue distribution, remain undefined; without these data, translation to human dosing regimens is speculative. Lastly, while SINAP was well tolerated in vitro, in vivo toxicity screens are needed to rule out off-target effects, particularly given the relatively high micromolar concentrations employed for dissolution.

Future investigations should therefore pursue a multi-tiered agenda: validation of its efficacy in lithogenic-diet rodent models with quantitative imaging of the stone burden; time-resolved pharmacokinetic profiling and formulation optimization, perhaps via solid-lipid nanoparticles or nano-emulsions to enhance the intestinal absorption; combinatorial studies pairing low-dose UDCA or ezetimibe with SINAP to test for additive or synergistic outcomes; and finally, system-level analyses—transcriptomic, metabolomic, and microbiome—to map the broader metabolic repercussions and uncover gut–liver axis interactions. Such work will not only refine our mechanistic understanding but also inform rational clinical trial design.

## 4. Materials and Methods

### 4.1. Chemicals and Reagents

The following reagents were utilized for cell culture: Dulbecco’s Modified Eagle’s Medium (DMEM) (Gibco, Thermo Fisher Scientific, Waltham, MA, USA), fetal bovine serum (FBS) (Sigma-Aldrich, St. Louis, MO, USA), penicillin–streptomycin solution (Thermo Fisher Scientific, Waltham, MA, USA), Trypsin–EDTA (0.25%) (Gibco, Thermo Fisher Scientific, Waltham, MA, USA), and Phosphate-Buffered Saline (PBS, pH 7.4) (Sigma-Aldrich, St. Louis, MO, USA). The HepG2 cell line (human hepatocellular carcinoma) was obtained from the American Type Culture Collection (ATCC, Manassas, VA, USA). Cells were cultured in Dulbecco’s Modified Eagle Medium (DMEM; Gibco, Waltham, MA, USA) supplemented with 10% fetal bovine serum (FBS; Gibco, Waltham, MA, USA) and 1% penicillin–streptomycin (100 U/mL penicillin and 100 µg/mL streptomycin) and maintained at 37 °C in a humidified atmosphere of 5% CO_2_. The cells were passaged when confluency reached approximately 80–90%. All experiments were performed using cells between passages 8 and 12 to ensure the consistency and reproducibility of the results [27]. The test compounds included sinapic acid (SINAP) (Sigma-Aldrich, St. Louis, MO, USA) and ezetimibe (EZE) (Sigma-Aldrich, St. Louis, MO, USA). Dimethyl Sulfoxide (DMSO) (Sigma-Aldrich, St. Louis, MO, USA) was used as the solvent.

### 4.2. The Cytotoxicity Assay (MTT Assay)

The cytotoxicity of sinapic acid (SINAP), ezetimibe (EZE), and ursodeoxycholic acid (UDCA) was evaluated using the MTT assay, a colorimetric technique that assesses mitochondrial activity as a proxy for cell viability [28]. HepG2 cells were seeded into 96-well plates (Corning, New York, NY, USA) at a density of 1 × 10^4^ cells per well in 100 µL of complete DMEM and incubated at 37 °C in a 5% CO_2_ humidified atmosphere for 24 h to allow for adherence. The cells were then treated with varying concentrations of SINAP (2.5–200 µg/mL), EZE (0.5–100 µM), and UDCA (10–200 µg/mL) for 24 h. The controls received an equivalent volume of the vehicle (0.1% DMSO).

Following treatment, 20 µL of 5 mg/mL MTT solution (3-[4,5-dimethylthiazol-2-yl]-2,5-diphenyltetrazolium bromide; Sigma-Aldrich, St. Louis, MO, USA) was added to each well and incubated for 4 h at 37 °C. After incubation, the supernatant was gently aspirated, and 150 µL of DMSO was added to each well to dissolve the resulting formazan crystals. The absorbance was measured at 570 nm using a BioTek Synergy HTX ELISA plate reader (BioTek Instruments, Winooski, VT, USA). Cell viability was expressed as a percentage relative to that in the untreated control (100%). All experiments were performed in biological triplicate (*n* = 3), with each sample run in technical triplicate.

### 4.3. Real-Time PCR Analysis (qPCR)

A gene expression analysis of cholesterol-transporter-related genes (ABCG5, ABCG8, and LXRα) was included using quantitative real-time PCR. Total RNA was extracted from the treated HepG2 cells using TRIzol reagent (Thermo Fisher Scientific, Waltham, MA, USA) following the manufacturer’s protocol. RNA purity and concentration were determined using a NanoDrop™ spectrophotometer (Thermo Scientific, Waltham, MA, USA). Only RNA samples with an A260/A280 ratio between 1.8 and 2.0 were used for downstream applications.

First-strand cDNA was synthesized from 1 µg of total RNA using the GoScript™ Reverse Transcription System (Promega, Madison, WI, USA) in a 20 µL reaction volume. qPCR amplification was performed using SYBR Green Master Mix (Thermo Fisher Scientific, Waltham, MA, USA) in a StepOnePlus™ Real-Time PCR System (Applied Biosystems, Foster City, CA, USA). Each 20 µL reaction contained 10 µL of 2× SYBR Green PCR Master Mix, 1 µL each of the forward and reverse primers (10 µM), 2 µL of cDNA, and 6 µL of nuclease-free water.

The thermal cycling conditions were as follows: initial denaturation at 95 °C for 10 min, followed by 40 cycles of 95 °C for 15 s, 60 °C for 30 s, and 72 °C for 30 s. A melting curve analysis was performed to confirm the specificity of the amplicons. Each qPCR run included a non-template control. The relative expression of the target genes was normalized to β-actin (ACTB) as a housekeeping gene and calculated using the 2^−ΔΔCt^ method [29]

The primers used for gene expression analysis were

- β-Actin (ACTB)
  Forward Primer: 5′-CACCATTGGCAATGAGCGGTTC-3′;
  Reverse Primer: 5′-AGGTCTTTGCGGATGTCCACGT-3′.
- ABCG5
  Forward Primer: 5′-AGGCTCAGTTACAGGCTCAGAG-3′;
  Reverse Primer: 5′-GTCCCACTTCTGCTGGCATGAT-3′.
- ABCG8
  Forward Primer: 5′-ATGAACTGGAAGACGGGC-3′;
  Reverse Primer: 5′-TGAAGGGTCTGCTCAG-3′.

### 4.4. ELISA for Bile Acid Secretion

Bile acid secretion into the culture medium was quantified using a commercially available Human Total Bile Acid ELISA Kit (MyBioSource, San Diego, CA, USA; Cat. No: MBS2606192), according to the manufacturer’s instructions. The HepG2 cells were cultured and treated with SINAP (50 µg/mL), EZE (25 µM), or the vehicle for 24 h. After treatment, cell culture supernatants were collected and centrifuged at 3000 rpm for 10 min at 4 °C to remove debris.

Samples were either used directly or diluted (1:2) with the assay buffer depending on the expected bile acid concentrations. A 100 µL volume of standards or supernatants was added to the ELISA plate and incubated as per the kit protocol. The absorbance was measured at 450 nm using a BioTek ELISA plate reader (BioTek Instruments, Winooski, VT, USA). A standard curve was generated using known concentrations of bile acids (0–150 µmol/L), and the total bile acid concentration in the samples was interpolated from the curve. All measurements were performed in biological triplicate and run in technical duplicate [30].

### 4.5. Western Blot Analysis

Proteins were extracted using RIPA Cell Lysis Buffer (Boster Bio, Pleasanton, CA, USA; SKU: AR0105-100) and quantified using the Bicinchoninic Acid (BCA) Protein Assay Kit (Thermo Fisher Scientific, Waltham, MA, USA). Proteins were separated through SDS-PAGE, transferred onto PVDF membranes (Millipore, Burlington, MA, USA), and blocked using 10% BSA in TBST. The primary antibodies included anti-ABCG5 (Proteintech, Rosemont, IL, USA; Cat. No: 27722-1-AP), anti-ABCG8 (Proteintech, Rosemont, IL, USA; Cat. No: 24453-1-AP), and Rabbit GAPDH (Cell Signaling, Danvers, MA, USA; Cat. No: 2118). HRP-conjugated goat anti-rabbit IgG (H+L) (Thermo Fisher Scientific, Waltham, MA, USA; Cat. No: 31460) was used as the secondary antibody. Detection was performed using enhanced chemiluminescence (ECL) reagents (Pierce, Rockford, IL, USA).

### 4.6. In Vitro Gallstone Dissolution Activity

#### 4.6.1. Materials

Normal saline (0.9%), ethanol, 10% formalin, distilled water, and the standard drug ursodiol were used. Cholesterol levels were measured using the AUTOSPANR Liquid Gold Cholesterol Kit (SISCO, Mumbai, India)

#### 4.6.2. Gallstone Collection and Donor Criteria

Cholesterol gallstones were collected from five adult patients (aged 30–65 years) undergoing elective laparoscopic cholecystectomy for symptomatic gallstone disease at a tertiary-care hospital. The inclusion criteria involved patients diagnosed with pure cholesterol stones. The exclusion criteria included patients with pigment stones, mixed-type gallstones, liver disease, or metabolic disorders.

Gallstones were collected immediately post-surgery, rinsed with sterile saline, and stored in sterile containers at 4 °C. A total of 9 cholesterol stones were obtained and used in the dissolution assay. All samples were collected following the provision of informed written consent under approval from the Institutional Ethics Committee (SRM IEC No: 8777/IEC/2024). Informed consent was obtained from all subjects involved in the study. Additionally, tissue samples were collected post-surgery with the assistance of the physician, following patient consent and ethical guidelines.

#### 4.6.3. Methods

The gallstones were dried at 45 °C and weighed using an airtight electronic balance. They were then incubated in human bile and treated with sinapic acid (1 mg/mL and 2 mg/mL) or ursodiol (2 mg/mL) at 37 °C for four weeks. Weekly, the gallstones were dried and reweighed to assess the weight reduction. The amount of cholesterol released was measured using an autoanalyzer. Changes in gallstone weight and cholesterol release were evaluated to determine the efficacy of sinapic acid in comparison to that of the reference drug ursodiol [31].

### 4.7. Statistical Analysis

All experiments were performed in biological triplicate (*n* = 3), and the results are presented as the mean ± standard deviation (SD). Statistical significance was determined using a one-way analysis of variance (ANOVA) followed by Bonferroni’s post hoc multiple comparison test to evaluate differences among the treatment groups. The significance threshold was set at *p* < 0.05. The graphical and statistical data analysis was included using GraphPad Prism version 10.3 (GraphPad Software, San Diego, CA, USA). In all figures, *p* < 0.05 is considered statistically significant (*), *p* < 0.01 is considered highly significant (*), and *p* > 0.05 is considered not significant (ns). All datasets, including those for the cell viability, gene expression (qPCR), bile acid secretion (ELISA), protein densitometry (Western blot), and gallstone dissolution assays, were evaluated using this approach.

## 5. Conclusions

The data presented herein identify sinapic acid as a rapid-acting, mechanistically rational, and apparently safe phytochemical with the capacity to dissolve cholesterol gallstones while promoting hepatobiliary cholesterol efflux through LXRα-driven ABCG5/8 induction. By uniting computational predictions, cellular signaling, bile acid measurements, and physical dissolution into a cohesive narrative, this study lays a robust foundation for advancing SINAP from a laboratory proof-of-concept to a credible non-surgical therapeutic option in cholelithiasis. Given the global prevalence of gallstone disease and the limitations of the current pharmacotherapy and surgery, SINAP represents a promising addition to the therapeutic armamentarium, meriting expedited progression to in vivo and ultimately clinical evaluations.

## Figures and Tables

**Table 1:** IC_50_ values and cell viability ranges.

Treatment	Concentration Range	IC_50_ Value (µg/mL)	Max Viability (%)
SINAP	2.5–100 µg/mL	~28.03	~98
Ezetimibe	0.7–100 µM	~21.5	~94

**Table 2:** Fold change and statistical significance of transporter gene expression. ** *p* < 0.01.

Gene	Control	SINAP (50 µg/mL)	Ezetimibe (25 µM)	*p*-Value vs. Control
ABCG5	1.00	2.4 **	2.7 **	<0.01
ABCG8	1.00	1.9 **	2.1 **	<0.01

**Table 3:** Quantitative densitometry of protein expression.

Protein	Control	SINAP	Ezetimibe
ABCG5	1.00	2.1	2.3
ABCG8	1.00	1.8	2.0

**Table 4:** Summary of gallstone weight loss and cholesterol release.

Treatment	Gallstone Weight Reduction (%)	Cholesterol Released (mg/mL)
Control	0.0%	0.0
SINAP (1 mg/mL)	17.4%	0.28
SINAP (2 mg/mL)	30.6%	0.45
Ursodiol (2 mg/mL	34.2%	0.49

Notes: Comparison of gallstone weight reduction and cholesterol release into bile following a 4-week in vitro treatment with sinapic acid and ursodiol. Percent weight reduction was calculated from initial stone weights, and cholesterol content was measured using enzymatic assay kits. Sinapic acid showed dose-dependent dissolution and cholesterol solubilization comparable to those with ursodiol.

## Data Availability

The original contributions presented in this study are included in the article/Supplementary Materials. Further inquiries can be directed to the corresponding author.

## Supplementary Materials

The following supporting information can be downloaded at https://www.mdpi.com/article/10.3390/ph18091388/s1. Spreadsheets for in vitro MTT assay, Western blot images, and RT-PCR results were added.
