# Supplementary figures and images for: Sinapic Acid Regulates the LXRα–ABCG5/8 Axis in the Hepatocytes: A Potential Strategy for Cholesterol Gallstone Management
Source: Pharmaceuticals (Basel). 2025 Sep 17;18(9):1388. doi: 10.3390/ph18091388 (PMC12473006; doi:10.3390/ph18091388)

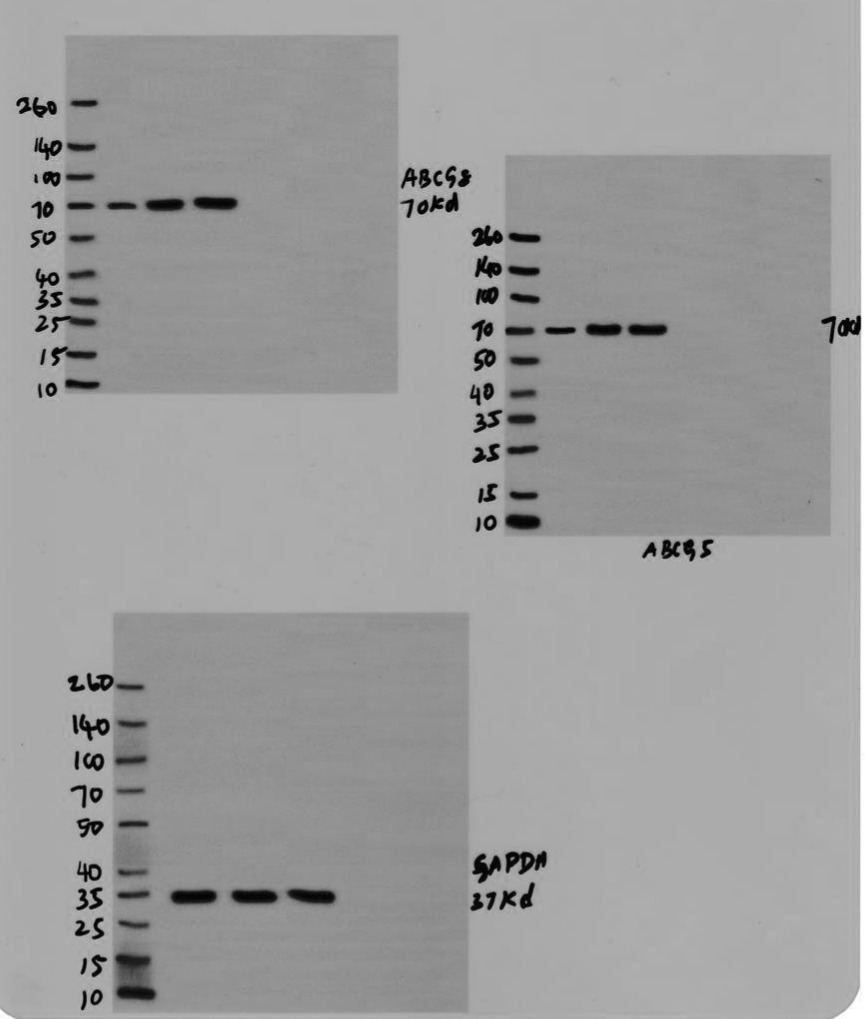

Supplement: Supplementary file 1 [file pharmaceuticals-18-01388-s001.zip › Western blot .JPG]
